# Bioinformatics analysis of disordered proteins in prokaryotes

**DOI:** 10.1186/1471-2105-12-66

**Published:** 2011-03-02

**Authors:** Gordana M Pavlović-Lažetić, Nenad S Mitić, Jovana J Kovačević, Zoran Obradović, Saša N Malkov, Miloš V Beljanski

**Affiliations:** 1 Faculty of Mathematics, University of Belgrade, P.O.B. 550, Studentski trg 16, 11001 Belgrade, Serbia; 2 Center for Information Science and Technology, Temple University, 303 Wachman Hall (038-24), 1805 N. Broad St., Philadelphia, PA 19122, USA; 3 Institute of General and Physical Chemistry, P.O.B. 551 Studentski trg 16, 11001 Belgrade, Serbia

## Abstract

**Background:**

A significant number of proteins have been shown to be intrinsically disordered, meaning that they lack a fixed 3 D structure or contain regions that do not posses a well defined 3 D structure. It has also been proven that a protein's disorder content is related to its function. We have performed an exhaustive analysis and comparison of the disorder content of proteins from prokaryotic organisms (i.e., superkingdoms Archaea and Bacteria) with respect to functional categories they belong to, i.e., Clusters of Orthologous Groups of proteins (COGs) and groups of COGs-Cellular processes (Cp), Information storage and processing (Isp), Metabolism (Me) and Poorly characterized (Pc).

We also analyzed the disorder content of proteins with respect to various genomic, metabolic and ecological characteristics of the organism they belong to. We used correlations and association rule mining in order to identify the most confident associations between specific modalities of the characteristics considered and disorder content.

**Results:**

Bacteria are shown to have a somewhat higher level of protein disorder than archaea, except for proteins in the Me functional group. It is demonstrated that the Isp and Cp functional groups in particular (L-repair function and N-cell motility and secretion COGs of proteins in specific) possess the highest disorder content, while Me proteins, in general, posses the lowest. Disorder fractions have been confirmed to have the lowest level for the so-called order-promoting amino acids and the highest level for the so-called disorder promoters.

For each pair of organism characteristics, specific modalities are identified with the maximum disorder proteins in the corresponding organisms, e.g., high genome size-high GC content organisms, facultative anaerobic-low GC content organisms, aerobic-high genome size organisms, etc. Maximum disorder in archaea is observed for high GC content-low genome size organisms, high GC content-facultative anaerobic or aquatic or mesophilic organisms, etc. Maximum disorder in bacteria is observed for high GC content-high genome size organisms, high genome size-aerobic organisms, etc.

Some of the most reliable association rules mined establish relationships between high GC content and high protein disorder, medium GC content and both medium and low protein disorder, anaerobic organisms and medium protein disorder, Gammaproteobacteria and low protein disorder, etc. A web site *Prokaryote Disorder Database *has been designed and implemented at the address http://bioinfo.matf.bg.ac.rs/disorder, which contains complete results of the analysis of protein disorder performed for 296 prokaryotic completely sequenced genomes.

**Conclusions:**

Exhaustive disorder analysis has been performed by functional classes of proteins, for a larger dataset of prokaryotic organisms than previously done. Results obtained are well correlated to those previously published, with some extension in the range of disorder level and clear distinction between functional classes of proteins. Wide correlation and association analysis between protein disorder and genomic and ecological characteristics has been performed for the first time. The results obtained give insight into multi-relationships among the characteristics and protein disorder. Such analysis provides for better understanding of the evolutionary process and may be useful for taxon determination. The main drawback of the approach is the fact that the disorder considered has been predicted and not experimentally established.

## Background

As a result of a growing number of experimental data on protein structure determination, it became evident that a significant number of proteins, under physiological conditions, do not possess a well defined 3 D ordered structure. They exhibit a variety of conformational isomers in which the atom positions and the polypeptide backbone (φ and ψ torsion angles) of the Ramachandran plot vary over time, with no specific equilibrium values, typically involving non-cooperative conformational changes [1]. Currently, they are known by different names, such as: "natively disordered/unfolded/denatured proteins", or "intrinsically disordered/unfolded/unstructured proteins", or "rheomorphic proteins", with the most frequently used term being "intrinsically disordered proteins (IDP)" and are recently reviewed in detail in [2-12]. In this paper we will use the term "disordered proteins" (DP). They may be completely disordered, or may be composed of both ordered and disordered regions of various lengths. In the DisProt DB, which is based on published experimental data on protein disordered regions in their native state, currently (May, 2010) there are 517 such proteins deposited, originated from various organisms. The length of these proteins varies between 38 and 3163 amino acids (AA) and length of their disordered regions is between 1 and 1480 AA. Out of all, 89 proteins are completely disordered and have length in the range 44 to 1861 AA [1,13]. On the basis of experimental and predictive data, some authors divided the disordered regions, according to the length (L), into three groups ((a) short: L = 4-30 AA, (b) long: L = 31-200 AA, (c) very long: L > 200 AA residues [14]), or five groups (L = 1-3, 4-15, 16-30, 31-100 and L > 100 AA residues [15]). Ward J. J. et al. [16] used the DisoPred2 disorder predictor and grouped *S. cerevisiae *proteins into three classes: (1) highly ordered proteins containing 0-10% of the predicted disorder, (2) moderately DP with 10-30% predicted disordered residues, and (3) highly DP containing 30-100% of the predicted disorder. Finally, fully DPs represent a special group of proteins of various lengths.

There is, however, no commonly agreed definition of protein disorder. The structural variability of DPs, same as their length, is high, ranging (by increasing level of order), from completely unstructured random coils (which resemble the highly unfolded states of globular proteins) to pre-molten globules (extended partially structured forms), or molten globules (compact disordered ensembles that may contain significant secondary structure), as proposed by protein trinity structure [17], or the protein-quartet [18] hypothesis. Any of these states may be the native state-that is, the state relevant to a protein's biological function. Some DPs can undergo a disorder-to-order, or vice versa, transition upon interaction with other molecules, whereas others remain substantially disordered during their action. In accordance to arising function, they are classified into, at least, 16 structural/functional categories, as listed in the DisProt database [12,18].

At the primary structure level, DPs are characterized by low sequence complexity (i.e. consist of repetitive short fragments) and are biased toward polar and charged, but against bulky hydrophobic and aromatic AA residues. Using a Composition Profiler [19], DPs were shown, based on AA composition, to be enriched in Ala, Arg, Gly, Gln, Ser, Glu, Lys and Pro and depleted in order-promoting Trp, Tyr, Phe, Ile, Leu, Val, Cys, Asn [5,20,21].

Using the TOP-IDP scale, based on AA properties such as hydrophobicity, polarity, volume, etc, Campen et al. [21] provided new ranking tendencies of AA from order to disorder promoting: Trp, Phe, Tyr, Ile, Met, Leu, Val, Asn, Cys, Thr, Ala, Gly, Arg, Asp, His, Gln, Lys, Ser, Glu, Pro. This new scale is qualitatively consistent with the previous one.

Experimentally, DPs may be detected by more than 20 various biophysical and biochemical techniques: x-ray diffraction crystallography, heteronuclear multidimensional NMR, circular dichroism, optical rotatory dispersion, Fourier transformed infrared spectroscopy, Raman optical activity, etc. Since DPs are difficult to study experimentally, because of the lack of unique structure in the isolated form [9,18], a number of prediction tools have been developed [22].

Programs for DP predictions may be grouped into two groups according to the principle of their operation: (1) those based on physicochemical properties of amino acids in proteins (PONDR family of disorder predictors, that include, among others: VL-XT, VL3, VSL1 and VSL2, FoldUnfold, PreLINK, IUPred, GlobProt, FoldIndex, etc.) and (2) those based on alignments of homologous protein sequences (RONN, DISOPRED) [9,11,23,24].

Taxonomically, DPs are represented in the proteomes of all of the three superkingdoms (Archaea, Bacteria and Eukarya). First results showed that at least 25% of the sequences in SwissProt DB contain long disordered regions [25,26].

Predicted to-be-disordered segments using the "Predictor of natural protein disorder" (PONDR), on a limited number of sequenced genomes, for archaea (7 genomes), vary in the ranges 9 - 57%, 9 - 37% and 4 - 24%, for segments length (L) ≥30, ≥40 and ≥50 AA, respectively, and for bacteria (22 genomes) in the ranges 13 - 52%, 6 - 33% and 2 - 21%, for segments L ≥30, ≥40 and ≥50 AA, respectively. For Eukarya (5 genomes) predicted ranges were significantly higher, i.e., 48-63%, 35-51% and 25-41%, for L ≥30, ≥40 and ≥50 AA, respectively [27]. In a subsequent analysis the same authors obtained somewhat different (larger) values regarding different predictors, genomes and the number of genomes used. For long disordered regions (> 40 AA) using the VL2 predictor, the percentage of disorder varies between 26 and 51% in archaea (6 genomes), with an average of 36%, 16 and 45% in bacteria (18 genomes), with an average of 28% and 52-67% in Eukarya, with an average of 60% [28]. Using the DISOPRED2 disorder predictor by Ward J. J. et al. [29], for a similar number of genomes, the authors showed that for archaea (6 genomes) the percentage of chains with contiguous disorder vary in range between 0.9-5.0% and 0.2-1.9% for segments L >30 and L >50 AA, respectively. For bacteria (13 genomes) the percentage of chains with contiguous disorder vary in range between 1.8-6.4% and 0.5-3.3% for L >30 and L >50 AA, respectively. For Eukarya (5 genomes) predicted values were also significantly higher: 27.5-36.6% and 15.6-22.1% for L >30 and L >50 AA, respectively.

The first analysis of the function of DPs on more than 150 proteins, with disordered regions L≥30 AA, from various species and under apparently native conditions, obtained by literature search, was performed by Dunker A. K. et al. [30]. They identified 28 separate biochemical functions, for 98 out of 115 disordered regions, that include protein-protein and protein-nucleic acids binding, protein modification, etc. Based on mode of action they proposed DPs classification into, at least, four classes: (1) molecular recognition, (2) molecular assembly/disassembly, (3) protein modifications and (4) entropic chain activities i.e., activities dependent on the flexibility, bendiness and plasticity of the backbone [1,5,30,31].

Xie H. et al. and Vučetić S. et al. [32,33], performed an analysis on approximately 200 000 proteins longer than 40 AA obtained from SwissProt DB, for disordered regions L≥40 AA using the VL3E predictor. The application revealed that out of 710 SwissProt keywords grouped into 11 functional categories (such as: biological process, molecular function, cellular components, etc.), 238 were associated with DPs, 310 were associated with ordered proteins and 162 gave ambiguity in function-structure associations. Both analyses concluded that DP's functions are prevalent in signaling and regulatory molecules and arise either from interactions between disordered regions and their partners from unfolded to folded form (molecular recognition and assembly/disassembly, protein modifications), or directly from the unfolded state (linkers, spacers, clocks) [20].

DPs are involved in key biological processes including signaling, recognition, regulation and cell cycle control, i.e., they may be further subdivided into more than 30 functional subclasses, as proposed by Dunker A. K. et al. [1,30].

Concerning taxonomic distribution of DPs, homologous (conserved sequences) analysis by Chen J. W. et al. [34,35] was performed, using data from UniProt and InterPro databases, for searching conserved predicted disorder by multiple sequence alignment. They found (a) that some predicted disordered regions are conserved within protein families, (b) that disorder may be more common in bacterial and archaeal proteins than previously thought, but (c) this disorder is likely to be used for different purposes than in eukaryotic proteins, as well as occurring in shorter stretches of protein domains [34,35].

Several DPs were experimentally shown to be associated with various diseases such as cancer and neurodegenerative diseases, while bioinformatics analyses revealed that many of them are associated with maladies such as cancer [36], diabetes [37], cardiovascular [38] and neurodegenerative diseases [39].

It is interesting to note peculiar reactions of DPs to environmental conditions such as temperature, pH, presence of counter ions, etc. DPs possess the so-called "turned out" response to heat, i.e., a temperature increase induces the partial folding instead of unfolding typically observed for ordered globular proteins. The effect is explained by the increased strength of hydrophobic interaction at higher temperatures that results in a stronger hydrophobic attraction, the major protein folding driving force [40]. Similarly, changes of pH (increase/decrease) and the presence of counter ions induce partial folding of DPs due to decreasing charge/charge molecular repulsion and permit stronger hydrophobic force leading to partial folding [40].

Since amino acid usage reflects, via codons, the genome GC value, it is possible to consider DPs abundance with respect to GC value. High GC value results in increased propensity of Gly, Ala, Arg and Pro, while low GC value is enriched with Phe, Tyr, Met, Ile, Asn and Lys [41,42]. Since Gly, Ala, Arg and Pro are overrepresented in disordered regions of proteins, it is expected that high genome GC values result in significant increase in DPs.

Other organism characteristics such as genome size, oxygen utility, optimum growth temperature, etc, may also be related to protein disorder through genome GC value and amino acid usage [41]. For example:

(a) It has been demonstrated, for some bacterial families, that there exists a relationship between genome size and GC level for aerobic, facultative anaerobic, and microaerophilic species, but not for anaerobic prokaryotes [41,43,44]. As compared to anaerobic, aerobic prokaryotes have shown increased GC content [45].

(b) In free-living organisms, larger genomes (more than 3 Mb), as a result of more complex and varied environments, show a trend toward higher GC content than smaller ones, while nutrient limiting and nutrient poor environments dictate smaller genomes of low GC [46].

(c) As it concerns optimum growth temperature, it has been noticed that genome and proteome contents of many thermophiles are characterized by overrepresentation of purine bases (i.e. A and G) in coding sequences, higher GC-content of their RNAs, change in protein amino acid physico-chemical properties, etc. On the other hand, proteins from thermophiles generally have more stable folds (more order) than proteins from mesophilic [47].

The goal of this study was twofold: first, to examine the relation of DPs of archaeal and bacterial proteomes to their function, i.e., Clusters of Orthologous Groups (COG) of proteins; second, to investigate the level of DPs in relation to various genomic, metabolic and ecological characteristics of organisms analyzed.

## Methods

### Dataset

The dataset includes all the proteins from organisms in the superkingdoms Archaea and Bacteria that contain annotated COGs of proteins: 25 (out of 64) archaea and 271 (out of 859) bacteria (Entrez Genome Project database, as of November 20th 2009), as well as taxonomic, genome and other organism information [48]. Superkingdom Archaea includes 3 phyla, Bacteria 17 phyla. Functional categories (25 categories) of proteins as defined in the COG of proteins database, and designated by the letters (function codes), may be classified, according to similar biological functions, into 4 groups: (1) Information storage and processing (Isp) (consisting of 5 categories: RNA processing and modification - A, Chromatin structure and dynamics - B, Translation, ribosomal structure and biogenesis - J, Transcription - K and DNA replication, recombination, and repair - L), (2) Cellular processes (Cp) (10 categories: Cell division and chromosome partitioning - D, Posttranslational modification, protein turnover, chaperones - O, Cell envelope biogenesis, outer membrane - M, Cell motility and secretion - N, Signal transduction mechanisms - T, Intracellular trafficking and secretion - U, Defense mechanisms - V, Extracellular structures - W, Nuclear structure - Y and Cytoskeleton - Z) (3) Metabolism (Me) (8 categories: Energy production and conversion - C, Carbohydrate transport and metabolism - G, Amino acid transport and metabolism - E, Nucleotide transport and metabolism - F, Coenzyme transport and metabolism - H, Lipid metabolism - I, Inorganic ion transport and metabolism - P and Secondary metabolites biosynthesis, transport and catabolism - Q) and (4) Poorly characterized (Pc) (2 categories: General function prediction only - R and Function unknown - S) [49]. Proteins not assigned to COGs are coded as N.C.

Although only about one third of the sequenced prokaryotes are COG-annotated (271 out of 859 Bacteria, 25 out of 64 Archaea), in the COG-annotated organisms all the phyla are represented, with number of organisms between 10% and 100% of all the sequenced genomes.

### Web site

The web site *Prokaryote Disorder Database *has been designed and implemented at http://bioinfo.matf.bg.ac.rs/disorder. The site contains complete results of the analysis of protein disorder performed for 296 completely sequenced prokaryotic genomes. There is a page specifically designed to provide the additional data to this paper http://bioinfo.matf.bg.ac.rs/disorder/paper.2010.wafl. That page contains a list of enumerated links. Wherever we reference a web site content in this paper, we specify an appropriate link at this page For example, in order to see detailed numerical characteristics of the dataset, the page *L1 *should be visited, which means that the page http://bioinfo.matf.bg.ac.rs/disorder/paper.2010.wafl should be opened and then the link „L1 - Basic numerical characteristics of the dataset” should be followed.

### Number of proteins by superkingdoms, phyla and COGs of proteins

The total number of proteins in proteomes of archaea and bacteria is 55815 and 754456, respectively. The number of proteins is the highest in the Metabolism group of COGs in both superkingdoms: 15718 (28%) in archaea and 222438 (29%) in bacteria. Among all the COGs of proteins, poorly characterized COG R is the largest in both superkingdoms, with 6819 and 69322 proteins, respectively (the largest portion is in the phylum Gammaproteobacteria). COG Y is empty; COGs W, Z are almost empty (1 protein in archaea and 57 in bacteria in W; 0 in archaea and 50 in bacteria in Z).

Phylum Gammaproteobacteria contains the largest number of proteins (229209 total). It is important to notice that, although there may be multiple occurrences of the same protein in the dataset (e.g., the same protein in more than one COG of proteins), numbers presented refer to different proteins in the collection considered (superkingdom, phylum, COGs, functional group of COGs, etc.). Thus, the number of proteins in a functional group of COGs does not have to be equal to the sum of numbers of proteins in each of the COGs belonging to that functional group. The same holds for other aggregates like average or standard deviation. There are 53689 (about 7%) non-unique proteins with 60779 extra occurrences. For the complete data see the web site, link *L1*.

### Number of proteins by length

Distribution of proteins by length in archaea and bacteria is represented on the web site (link *L2*). For proteins of length ≤ 1000 AA, the average protein length is 279 AA in archaea and 297 AA in bacteria.

### Number of proteins by length and COGs of proteins

Ranked by length and COGs of proteins, the number of proteins is the largest for lengths between 200 AA and 300 AA in COG R for both superkingdoms: 2044 proteins in Archaea, 22748 proteins in Bacteria. Number of proteins is the largest for the Metabolism group of COGs, as compared to other groups, for all lengths starting from 200 AA.

There are 10 proteins longer than 10000 AA, the longest being a non categorized protein from Bacteroidetes/Chlorobi, i.e., *Chlorobium chlorochromatii *CaD3 of L = 36805 AA.

### Organism information

For the dataset considered, five characteristics (genome size, GC content, habitat, oxygen requirement and temperature range), with two to five modalities each, have been downloaded from [48].

### Processing steps

1. A Perl program has been developed for downloading the protein sequences of archaeal and bacterial genomes.

2. Disorder predictors IUPred [50], VSL2, VSL2B, and VSL2P [51], have been compared based on the DisProt database [13]. A set of 10 proteins have been chosen with disordered regions determined by different experimental methods and the four predictors were applied to those proteins. Prediction quality measures (recall, precision, F-measure, sensitivity, specificity) have been calculated. Predictors from the VSL2 group gave similar results, better than IUPred, so we chose the fastest version (VSL2B). The VSL2B predictor was applied to all the proteins and disorder level was calculated for each amino acid occurrence.

3. A database has been designed and populated with taxonomic, COG of proteins, protein, disorder and organism info data

4. Programs in SQL and Java have been developed for analyses of COGs disorder contents:

• Analysis of *disordered regions*. Distributions of disordered regions of different length (≥ 1, 11, 21, 31, 41 AA), by protein in populated COGs of proteins, per 100 AA, by protein length, by organisms, COGs and phyla have been calculated.

• Analysis of *disordered amino acids*. Percentages of disordered amino acids by protein length have been calculated, as well as the number and percentage of amino acids in disordered regions of different length.

• Analysis of *proteins with disordered regions*. The number and percentage of proteins with disordered regions in COGs of proteins and phyla or superkingdoms, as well as the number and percentage of such proteins by protein length, have been analyzed.

5. Mole fractions for amino acids have been calculated for COGs of proteins (in superkingdoms and phyla) as well as fractional difference between disordered and ordered sets of regions for COGs. The mole fraction for the j-th amino acid (j = 1,20) in the i-th sequence (e.g., i-th protein in a given COG) is determined as P_j _= sum(n_i_*P_ji_)/sum(n_i_), where n_i _is the length of the i-th sequence and P_ji _- frequency of the j-th amino acid in the i-th sequence. The fractional difference is calculated by the formula (P_j_(a) - P_j_(b))/P_j_(b), where P_j_(a) is the mole fraction of the j-the amino acid in the set of predicted disordered regions in proteins of a given COG category (set a), and P_j_(b) is the corresponding mole fraction in the set of predicted ordered regions in proteins of the same COG category.

6. The obtained results have been grouped and analyzed by functional groups of COG categories.

7. Disorder contents have been analyzed for proteins in specific subsets of archaea and bacteria, based on some structural, morphological and ecological characteristics of organisms: genome size, GC content, oxygen requirement, habitat and optimal growth temperature.

a. Distribution of genome size in prokaryotes, calculated by Koonin et al. [52], clearly separates two broad genome classes with the 4 Mega base (Mb) border. We recalculated this distribution on superkingdoms Archaea and Bacteria and confirmed their classification in two modalities: "short" genome size (length < 4 Mb) and "long" genome size (length > 4 Mb) bacterial genomes (for archaea, 2.5 Mb).

b. Average GC content of bacterial genomes varies in range from 25% to 75% [46]. We considered three modalities for GC content: low, medium and high GC content, with borders at average GC content +/- one standard deviation.

c. We considered five modalities for habitat, found in the Entrez Genome Database [48]: aquatic, multiple, specialized (e.g., hot springs, salty lakes), host-associated (e.g., symbiotic) and terrestrial.

d. Most bacteria were placed into one of four groups based on their response to gaseous oxygen [48] - aerobic, facultative anaerobic (facultative for short), anaerobic and microaerophilic.

e. Based on temperature of growth archaea and bacteria were classified into the following modalities: mesophile and extremophile, i.e., thermophile, hyperthermophile and cryophile (or psychrophile).

The number of organisms for each modality of these characteristics in the dataset considered is presented on the web site (link *L10*). We analyzed correlations among different modalities of specific characteristics of organisms and disorder level in proteins of those organisms, and extended the study to multiple characteristics/disorder level correlations.

8. The independent-samples t-test has been used for testing deviation of disorder mean values among categories considered. Normality of the variables under analysis has been tested using the one-sample Kolmogorov-Smirnov test.

9. We applied algorithms for association rule mining in order to identify the most promising associations between the characteristics considered and disorder level [53]. Rules considered have the form A ⇒ B where A and B are sets of elements (*items*) represented in the data set. A is called the *body *of the rule, and B - the *head *of the rule. *Support *and *confidence *were primary quality measures of the rules considered in our experiments. Support reflects frequency of a set of items. Support for the rule A⇒B denoted by *s*(A⇒B), is defined as

$$s(\text{A}\Rightarrow \text{B})=\sigma \frac{\left(A\cup B\right)}{N}$$

where σ(X) denotes number of occurrences of an item X, and N - the total number of items. Confidence measures how often item B occurs when item A occurred, and for a rule A⇒B, it is defined as

$$c(\text{A}\Rightarrow \text{B})=\frac{\sigma (A\cup B)}{\sigma (A)}$$

The higher the confidence and support, the more reliable the rule is. In certain cases an anomaly arises where both support and confidence are very high but the rule itself does not give a useful result. Because of that, additional measures were used to estimate a rule's quality. One of them is *Lift*: for the rule A⇒B, it is calculated as *Lift*(A⇒B) = c(A⇒B)/s(B). If A and B are statistically independent, then Lift = 1. In case Lift > 1, A and B are said to be positively correlated, while in case Lift < 1, A and B are said to be negatively correlated. In this context, positive correlation means that the element B (in the head of the rule) is more frequent when A (body of the rule) occurred, than when A did not occur. Analogous holds for negative correlation. We used the IBM Intelligent Miner, which is a part of the programming package IBM InfoSphereWarehouse V9.5 (and later versions) [54]. It consists of three components: *Modeling*, used for model creation, *Scoring*, used for testing rules applied to new data in order to estimate benefits, and *Visualization*, used for presentation of results obtained. Modeling uses an *a priori *algorithm to mine association rules. Visualization enables fast detection of the rules that stand out. For bacteria in general, most of the genomes are mesophilic in temperature (more than 92%), so almost all the rules involve this element in the rule body or rule head. On the other side, most archaea (with COGs of proteins) are in Euryarchaeota, so most rules for archaea involve this phylum. Thus we chose only rules that conform to the following criteria:

• contain Euryarchaeota phylum neither in rule body nor in rule head

• contain modality mesophilic for the temperature attribute for bacteria, neither in rule body nor in rule head

• contain no more than two items in rule body

• minimum rule body, i.e., rules do not have rule body that is a superset of another rule body with the same rule head (except in case of more reliable rules)

• contain disorder attribute either in rule body or in rule head

• keep rules with as high confidence, support and lift parameters as possible

• of all the rules having the same head and different modalities of the same characteristics in the body, keep the most reliable.

## Results and discussion

Complete results of the analysis of disorder content - the number and percentage of disordered regions of various lengths, amino acid content of disordered regions, number and percentage of proteins containing disordered regions for 296 prokaryotic completely sequenced genomes can be found on the web site http://bioinfo.matf.bg.ac.rs/disorder. Here, we will present only the most important ones.

### Disorder content

Table 1 captures data about disordered and ordered regions of length ≥41 AA for proteins that contain such regions and for all the proteins in the dataset.

**Table 1:** Disordered and ordered regions of length ≥41 AA.

Super-kingdom	Type	AA Region	#regions/100 AA	% of proteins length	Avg. protein length	#regions/100 AA in all proteins	% of all proteins length	Avg. protein length (all proteins)
Archaea	disord	68,34	0,33	22,47	385,17	0,09	6,04	290,11
	order	88,89	0,74	65,85	311,62	0,71	63,14	290,11

Bacteria	disord	73,88	0,31	23,10	421,38	0,09	6,94	313,34
	order	88,95	0,73	65,24	339,70	0,70	62,62	313,34

The average length of each type of region is presented (AA/Region), followed by data for proteins containing such regions (number of regions per 100 AA, percentage of proteins length included in such regions, average protein length), followed by the corresponding data for all the proteins in each superkingdom.

It can be seen that proteins containing disordered regions of length ≥ 41 AA are (on average) significantly longer than an average protein in the whole dataset (33-34%, p-value < 0.001 for random samples of 5% of protein sets, using the independent-samples t test for mean values). Similarly, the number of disordered regions of length ≥ 41 AA per 100 AA is significantly higher for proteins containing such regions than for all the proteins (p-value < 0.001), while the corresponding number in proteins containing ordered regions is almost equal to that in all the proteins, meaning that almost all the proteins contain ordered regions of given length and only a small portion of them contain disordered regions of given length. The same relations hold for other region lengths.

If we take into account only proteins that are 'pure' (i.e. completely, by predictor) disordered or ordered, the results obtained are represented in Table 2. It can be seen that such proteins have smaller average length than proteins with mixed contents.

**Table 2:** Number and average length of fully disordered or ordered proteins.

Super-kingdom	Type	No. of proteins	Average protein length (AA/region)
Archaea	disord	450	88,23
	order	27	136,85

Bacteria	disord	7045	90,73
	order	408	124,50

A protein is considered fully disordered if all of its AA are predicted to be disordered (by predictor)

Percentages of proteins with disordered contents >90% ranges, by phyla, from 1.06% to 6.71% (except for phyla with less than 100 such proteins), while the phylum Planctomycetes has the largest percentage of 100% disordered proteins (5.22%), as presented in Table 3. The phylum Planctomycetes significantly deviates (p-value < 0.01) in both the percentage of proteins with > 90% disorder contents and in the percentage of 100% disordered proteins.

**Table 3:** Archaea and bacteria by phyla.

Super-kingdom	Phylum	#organisms	#proteins	#proteins > 90%	%proteins > 90%	#proteins = 100%	%proteins = 100%
Archaea	Crenarchaeota	5	12320	96	0,78	72	0,58
	Euryarchaeota	19	42960	614	1,43	424	0,99
	Nanoarchaeota	1	535	3	0,56	1	0,19

	Archaea total:	25	55815	713	1,28	497	0,89

Bacteria	Actinobacteria	19	63125	1240	1,96	868	1,38
	Alphaproteobacteria	32	75868	1122	1,48	796	1,05
	Aquificae	1	1527	10	0,65	7	0,46
	Bacteroidetes/Chlorobi	8	24573	260	1,06	205	0,83
	Betaproteobacteria	19	59834	1335	2,23	941	1,57
	Chlamydiae/Verrucomicrobia	10	10977	177	1,61	137	1,25
	Chloroflexi	2	3036	39	1,28	31	1,02
	Cyanobacteria	15	42933	559	1,30	395	0,92
	Deinococcus-Thermus	3	6556	121	1,85	86	1,31
	Deltaproteobacteria	7	24128	435	1,80	347	1,44
	Epsilonproteobacteria	7	12520	164	1,31	122	0,97
	Firmicutes	72	175762	2565	1,46	1944	1,11
	Fusobacteria	1	2064	23	1,11	15	0,73
	Gammaproteobacteria	67	229209	2608	1,14	1982	0,86
	Planctomycetes	1	7322	491	6,71	382	5,22
	Spirochaetes	6	13166	170	1,29	135	1,03
	Thermotogae	1	1856	26	1,40	23	1,24

	Bacteria total:	271	754456	11345	1,50	8416	1,16

Number of organisms, number of proteins, number and percentage of proteins with >90% disordered amino acids, number and percentage of 100% disordered proteins.

### Number of disordered regions

**Figure 1:**
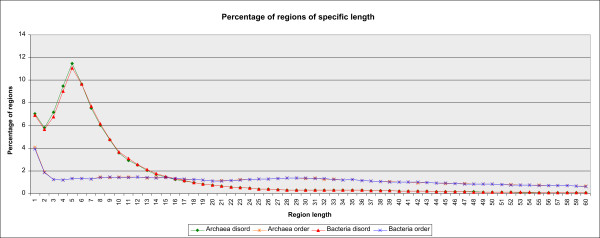
**The percentage of disordered and ordered regions of specific length in archaea and bacteria**. The percentage is calculated relative to the total number of regions of the corresponding type (disordered, ordered).

Comparison of archaea and bacteria based on the number of disordered (and ordered) regions gives almost no difference between these superkingdoms. The highest abundance of disordered regions have segments of length 1-10 AA, in all the phyla of Archaea and Bacteria. The next most frequent interval (11-20 AA) is about five times less populated, and it is, in turn, three to four times higher than the number of disordered regions in the interval 21 to 31 AA (see the web site, link *L3*). This similarity holds even if we decrease the interval length to one, as shown in Figure 1. Furthermore, similarity with this shape of curve (and corresponding percents) holds not only for phyla but even for single organisms, as shown on the web site.

Direct comparison of our results to those previously published [27-29] is not possible due to different methods (predictors) used, numbers of genomes analyzed and genomes themselves. For archaea (25 genomes), the percentage of disordered regions of L≥ 41 AA vary in range between 8% and 46%, as compared to 9 - 37% obtained by an early estimate by Dunker A. K. et al. [27]. For bacteria (271 genomes), the percentage of disordered regions of L≥ 41 AA vary in range between 8 and 53%, as compared to 6 - 33% obtained by Dunker A. K. et al. [27].

### Number of disordered regions by COGs of proteins

The average number of disordered regions (of L ≥1, 11, 21, 31, 41 AA) by protein and COG of proteins for archaea and bacteria, is presented on the web site (link *L4*). The average number of disordered regions of L ≥1 AA in all the proteins coded by COGs is 5.71 by protein. The largest average number of disordered regions is found in the proteins coded by COG L in archaea (7.41) and the proteins coded by COG V in bacteria (7.76), with the exception of poorly populated COGs W (17.77) and Z (11.28). For disordered regions of L ≥11, 21, 31, 41 AA, the average number of disordered regions is the highest for proteins coded by COG N, for both archaea and bacteria, again with the exception of poorly populated COGs W and Z. In general, the highest average number of disordered regions is found in proteins coded by COGs in the Cp functional group (COGs: D, M, N, O, T, U, V, W), followed by Isp (COGs: A, B, J, K, L), followed by Me (COGs: C, E, F, G, H, I, P, Q), followed by Pc (COGs: R, S). Proteins coded by genes N.C. have a low number of disordered regions of any length. The highest average number of disordered regions of L ≥11, 21, 31, 41 AA, by protein, in most phyla, is found in COGs N (Cp) and L (Isp).

The mean value of all the average numbers of disordered regions in proteins, by COGs, for regions of L ≥1 AA in bacteria is 6.91, with STD 2.55, so that COGs deviating more than 1STD from the mean value are W and Z (high average); the N. C. group of proteins significantly deviates with a low average. Archaea are much more stable: mean value is 6.05 with STD 0.79. For longer disordered regions, the only deviating COG in bacteria is W and in archaea the COGs K, L, T, V, P (higher average, see the web site, link *L4*).

### Number of disordered regions per 100 AA by COGs of proteins

**Figure 2:**
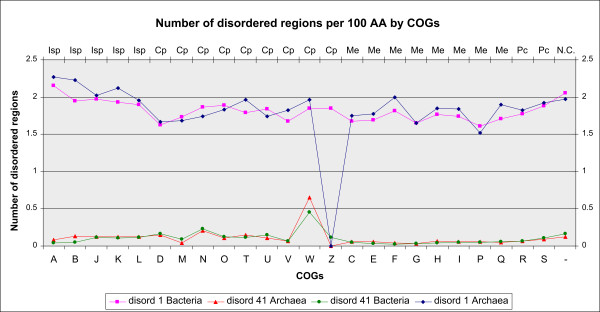
**The number of disordered regions per 100 AA, by COGs of proteins**. Ordering of COGs is by functional group: Isp Cp, Me Pc, NC. Disordered regions of L≥1 AA and L≥41 AA are represented for archaea and bacteria (disord1Archaea, disord41Archaea, disord1Bacteria, disord41Bacteria).

The average number of disordered regions per 100 AA by COGs of proteins neutralizes effects of protein length. It is depicted, for different lengths of disordered regions, in Figure 2. For bacteria, the average number of disordered regions of L ≥1 AA per 100 AA by COGs equals 1.82 with STD 0.13, while in archaea the corresponding values are 1.88, 0.18, respectively. Deviating COGs converge (over increasing length of disordered regions) to W and N in bacteria and just a singleton COG W (with just 1 protein) in archaea. Proteins classified in the functional group of Metabolism COGs, show again the lowest disorder. This suggests that distribution of disordered regions of unlimited length (≥1 AA) differs from those for longer regions so that regions of unlimited length may be abandoned.

### Number of disordered regions per 100 AA by protein length by COGs of proteins

**Figure 3:**
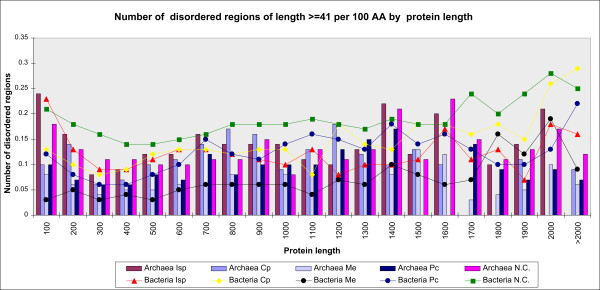
**The number of disordered regions per 100 AA, by protein length and functional groups of COGs of proteins**. All the proteins in the corresponding functional groups are considered. Disordered regions of L ≥41 AA are presented. Functional groups of archaea are represented by vertical bars, of bacteria by lines.

For disordered regions of L ≥41 AA, the average number of disordered regions per 100 AA by protein length in bacteria decreases up to the length of 300 AA, then steadily increases by all functional groups of proteins coded by COGs (Figure 3). Similar holds for archaea (see the web site, link *L5*, for the corresponding data about specific phyla and organisms). For proteins of length less than 1600 AA in Me COGs of proteins, disorder is consistently lower in bacteria than in archaea.

**Figure 4:**
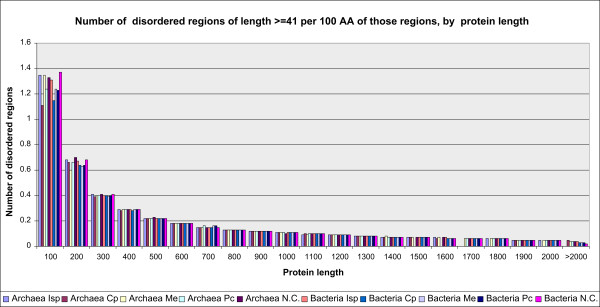
**The number of disordered regions per 100 AA of those regions themselves**. Disordered regions of L ≥41 AA, by protein length and functional groups of COGs, are presented. Functional groups of both archaea and bacteria are represented by vertical bars.

Figure 4 represents the number of disordered regions of L ≥41 AA per 100 AA of the regions themselves, by protein length and functional groups of COGs. The strict decreasing monotony for both archaea and bacteria and all the groups of COGs suggests that length of disordered regions increases monotonically with protein length.

### Amino acid contents of disordered regions

The average percentage of predicted-to-be-disordered amino acids is estimated to be 21.05% in archaea and 21.78% in bacteria. In disordered regions of L ≥11, 21, 31, 41 AA, the percentage is 14.07, 9.91, 7.91, 6.04, respectively for archaea, and 14.99, 10.08, 8.79, 6.94, respectively, for bacteria. For specific phyla see the web site data (link *L6*).

### The average percentage of disordered amino acids by protein

**Figure 5:**
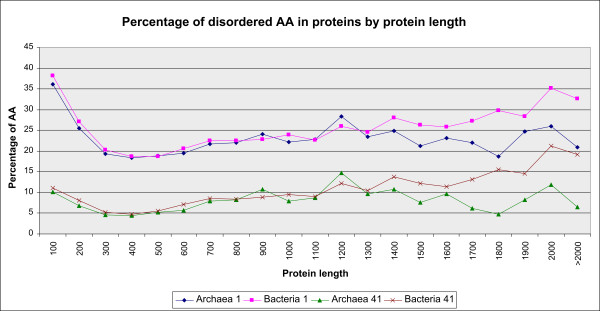
**The percentage of disordered amino acids in proteins by protein length**. Amino acids in disordered regions of L ≥1, 41 AA are presented for both archaea and bacteria.

The percentage of amino acids predicted to belong to disordered regions is the highest for proteins of length 0-100 AA in both archaea (about 36%) and bacteria (about 38%) for amino acids in unlimited length disordered regions; it then decreases to the minimum at 400 AA long proteins, stagnates to 500 AA at about 20% and then increases up to 1400 AA long proteins (Figure 5). The percentage is higher in bacteria than in archaea in all the intervals of protein length except for the interval 800-900 and 1100-1200 AA. In bacteria the average percentage of disordered AA has an upward peak at 1900-2000 AA long proteins of about 35%, while in archaea there is a downward peak at proteins consisting of 1700-1800 AA, of about 18%. Regarding tendency, similar holds for amino acids in longer disordered regions

### Proteins with disordered regions

**Figure 6:**
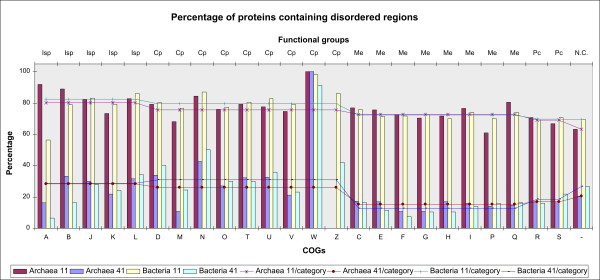
**The percentage of proteins containing disordered regions, by COGs of proteins and functional groups**. Disordered regions of L ≥ 11, 41 AA are presented. COG values are represented by vertical bars and functional group values are represented by lines.

The percentage of proteins containing disordered regions of L ≥1, 11, 21, 31, 41 AA, in archaea (bacteria), is around 99.9% (both), 71% (74), 43% (46), 30% (32), 20% (22), respectively. Distribution by COGs of proteins for regions of length L ≥ 11, 41 AA, is represented in Figure 6. Extremely high percentages of proteins with disordered regions of any length have the proteins coded by COG N and scarcely populated COG W, in the Cp category of COGs (see the web site, link *L7*). The percentage of proteins containing disordered regions of different length is consistently distributed among functional groups of COGs in both superkingdoms - the highest percentage is in the Cp and Isp categories, the lowest percentage is in the Me and Pc categories.

Among all the phyla, the highest percentage of proteins containing long disordered regions is found in Planctomycetes (about 63% in Cp, 54% in Isp, 39% in Pc, and 30% in Me categories), for which a number of genes have been found (through sequence comparisons) that are similar to genes found in eukaryotes [55]. The highest overall percentage of proteins with disordered regions of L ≥41 AA, by all the functional groups of COGs, in bacteria has the phylum Planctomycetes, about 40%, and the lowest - the phylum Chloroflexi, about 13%. Archaea is less populated with such proteins: 10-23% by phyla.

### Mole fractions and fractional differences

The overall statistics on amino acids - number and percentage of each in specific superkingdom and phylum, in disordered and ordered regions of specific length, is presented on the web site (link *L8*). Table 4 represents data about amino acids in all the proteins, in proteins containing disordered/ordered regions of L ≥ 41 AA, as well as in such regions themselves.

**Table 4:** Distribution of amino acids.

	Superkingdom Archaea			Superkingdom Bacteria		
		
AA	% in all proteins	% in proteins with disordered regions L > = 41	% in disordered region L > = 41	% in all proteins	% in proteins with disordered regions L > = 41	% in disordered region L > = 41
Ala	7,48	1,82	8,50	9,52	2,64	11,84
Arg	5,25	1,57	7,30	5,65	1,72	7,72
Asn	3,99	0,79	3,67	3,84	0,90	4,03
Asp	5,61	1,63	7,62	5,36	1,35	6,06
Cys	0,90	0,15	0,70	0,97	0,12	0,54

Gln	2,16	0,72	3,33	3,91	1,30	5,83
Glu	7,82	2,93	13,68	6,13	1,99	8,95
Gly	7,29	1,44	6,72	7,40	1,59	7,12
His	1,62	0,29	1,34	2,15	0,38	1,71
Ile	7,67	0,97	4,53	6,21	0,75	3,37

Leu	9,57	1,61	7,51	10,35	1,75	7,86
Lys	6,29	1,70	7,92	5,05	1,53	6,87
Met	2,32	0,42	1,98	2,41	0,44	1,98
Phe	4,08	0,44	2,07	4,02	0,42	1,87
Pro	4,15	1,13	5,28	4,44	1,46	6,54

Ser	6,08	1,79	8,34	6,00	1,91	8,56
Thr	5,09	1,38	6,45	5,36	1,38	6,20
Trp	1,03	0,09	0,42	1,24	0,10	0,45
Tyr	3,86	0,35	1,65	2,97	0,29	1,30
Val	7,76	1,24	5,79	7,04	1,09	4,88

The percentage of each amino acid, in all the proteins of archaea/bacteria, in proteins containing disordered regions of L≥41 AA, as well as in such regions themselves, is presented.

It can be noticed that, in both superkingdoms, amino acids Ala, Asp, Glu, Lys, Pro, Gln, Arg, Ser, Thr, are more represented in disordered regions than in the whole proteins, while amino acids Cys, Phe, Gly, His, Ile, Leu, Met, Asn, Val, Trp, Tyr, are less represented in disordered regions than in the whole proteins. This is in accordance with "order-promoting" property of amino acids Cys, Phe, His, Ile, Leu, Val, Trp, Tyr, and "disorder-promoting" property of polar and charged amino acids Ala, Glu, Lys, Pro, Gln, Arg, Ser [5,19-21].

**Figure 7:**
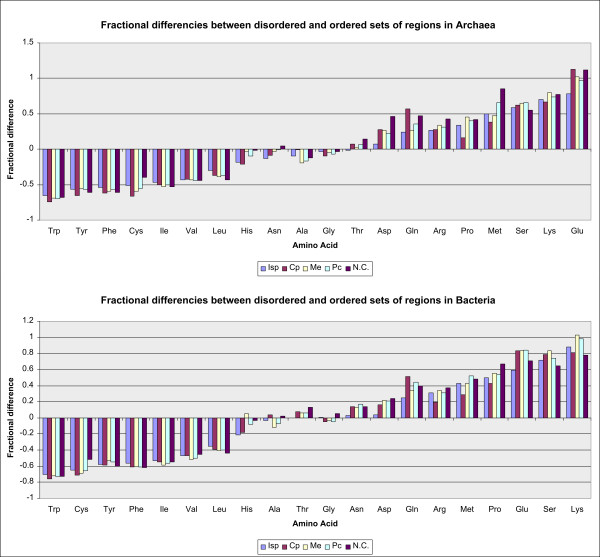
**Fractional differences in composition between disordered and ordered sets of regions**. Fractional differences by functional groups of COGs of proteins, for archaea and bacteria, are presented. x-axis represents amino acids; for a given amino acid, y-axis represents the fractional difference in composition between a set of disordered and a set of ordered regions in proteins, for each COG group - Isp, Cp, Me, Pc, N.C)

Figure 7 represents fractional difference for amino acids by functional groups of COGs of proteins, for archaea and bacteria. Ordering of amino acids is ascending with respect to mole fraction (fractional difference). Negative value for fractional difference of an amino acid corresponds to its low level of disorder, and positive value - high level of disorder. Results are again consistent with [5,19-21], showing that in both superkingdoms disorder is low in most of the so-called order-promoting amino acids and high in those known as "disorder-promoting". Fractional difference of Asn is inconsistent among archaea and bacteria, while disorder-promoting amino acids Gly and Ala are slightly more ordered. So the ascending ordering of amino acids with respect to fractional difference is close to Vihinen's flexibility scale [56].

### Ordering of COGs of proteins and functional groups

Different criteria analyzed so far for measuring the disorder content of proteins in specific COGs of proteins and COG groups gave different, yet comparable results that can be represented through ordering of COGs in ascending order of disorder based on these criteria (see the web site, link *L9*). It may be noticed that orderings for disordered regions of unlimited length deviate from all the others, thus being less reliable than orderings based on longer regions.

The following are sequences of COGs of proteins in ascending order of disorder contents (by different criteria), for disordered regions of L ≥41 AA (label "-" denotes "Not in COG" proteins):

**a) **Ascending order of average number (by protein) of disordered regions:

Archaea: F,G,M,Q,P,I,E,H,C,R,A,S,-,V,K,J,O,B,U,L,D,T,N,W

Bacteria: F,A,G,H,E,I,B,C,P,R,Q,S,V,K,J,M,-,O,T,L,U,D,Z,N,W

**b) **Ascending order of number of disordered regions per 100 AA:

Archaea: G,M,F,Q,P,E,I,C,H,R,V,A,S,O,U,K,L,-,J,B,T,D,N,W

Bacteria: F,G,E,A,H,I,C,B,P,Q,R,V,M,K,S,T,J,Z,L,O,U,D,-,N,W

**c) **Ascending order of percentage of proteins containing disordered regions:

Archaea: G,M,F,P,Q,I,A,E,R,S,H,C,-,V,K,O,J,L,T,U,B,D,N,W

Bacteria: A,F,G,H,E,I,P,R,B,Q,C,S,V,K,M,-,J,T,O,L,U,D,Z,N,W

**d) **Ascending order of average percentage of amino acids in disordered regions:

Archaea: G,M,F,Q,E,I,P,V,C,H,R,A,S,O,J,K,B,U,-,L,T,D,N,W,

Bacteria: F,G,E,H,I,B,P,C,Q,A,V,R,M,K,J,L,S,T,Z,O,U,-,N,D,W

**Figure 8:**
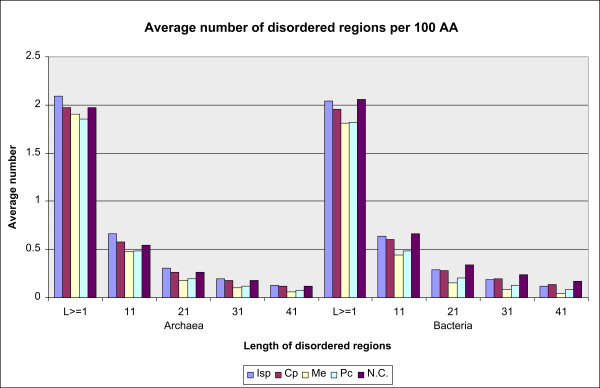
**Disordered regions per 100 AA, by region length and COG groups**. X-axis represents increasing disordered region length; the bar for a given region length and a given COG group represents the number of disordered regions of the given length, per 100 AA, in the corresponding functional group.

The overall conclusion of disorder analysis by COGs of proteins is that Isp (i.e., signaling, recognition, regulation, control) highly populated COGs (J, K, L), as well as most of (non-empty) Cp COGs (D, V, T, M, N, Z, U, O) show abundance of disorder (higher level than average), while Me COGs (C, G, E, F, H, I, P, Q) exhibit scarcity of disorder (lower level than average). Figure 8 illustrates this relationship between COGs in Isp, Cp and Me functional groups, when the number of disordered regions per 100 AA is considered. Similar relationships hold for other disorder criteria (see the web site, link *L9*).

### Organism info - genomic and ecological characteristics

Characteristics of superkingdoms and specific phyla with respect to disorder regions of L ≥1, 11, 41 AA, are represented on the web site (link *L10*). Table 5 presents GC content on superkingdom level with respect to the percentage of amino acids in disordered regions of L ≥41 AA, in different COG of protein groups.

**Table 5:** GC content with respect to disorder content.

			Functional group				
Superkingdom	GC content	Type	Isp	Cp	Me	Pc	**N.C**.
Archaea	Low	disord	5,80	4,87	1,82	2,36	6,86
		order	59,40	66,91	70,63	70,08	65,82
	
	Medium	disord	6,27	5,09	1,89	3,83	6,25
		order	59,79	65,61	71,26	66,92	62,23
	
	High	disord	16,97	16,56	9,00	10,91	17,48
		order	42,56	47,32	56,11	54,35	47,00

Bacteria	Low	disord	6,39	9,48	2,29	5,73	15,25
		order	60,57	60,07	69,33	64,75	50,14
	
	Medium	disord	6,31	7,40	1,80	4,87	10,41
		order	60,26	62,10	71,29	65,56	52,43
	
	High	disord	10,39	11,06	3,95	7,77	20,02
		order	56,92	59,49	69,33	62,89	46,97

The percentage of amino acids in disordered regions of L ≥41 AA is represented for each GC content level (low, medium, high) and each functional group of COGs (Isp, Cp, Me, Pc, N.C.) in archaea/bacteria.

**Figure 9:**
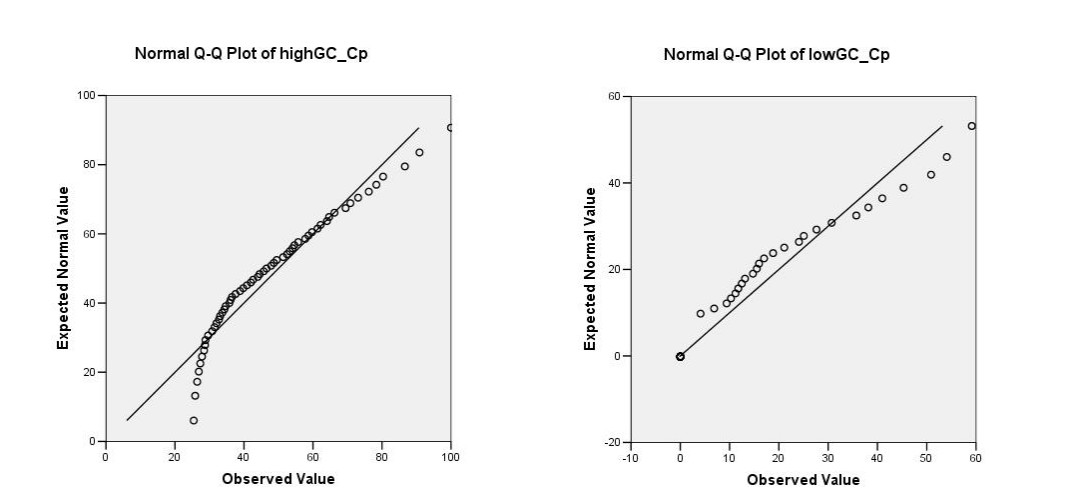
**Q-Q plot of a normality test**. Test variables are "disorder content in Cp protein category of archaea high GC organisms" (left) and "disorder content in Cp protein category of archaea low GC organisms" (right). Disorder is measured as a percentage of amino acids in long disordered regions (L≥41). Variables are reduced to 25% of the most disordered proteins.

The data in the table may be interpreted as follows:

In archaea, high GC organisms contain up to 300% more DPs than organisms with low and medium GC contents; independent-samples t-test statistically rejects hypothesis about equal means of disorder contents in each of the functional categories of high and low GC organisms as well as of high and medium GC organisms (p = 0.000). Such a hypothesis can not be rejected for low and medium GC organisms (p > 0.1). Variables involved in the test are reduced to 25% of the most disordered proteins in a given functional category (Isp, Cp, Me, Pc, N.C.), of high, low or medium GC organisms. The reduction is applied since a large portion of proteins in low and medium GC organisms are 0-disordered regarding large disordered regions (e.g., 85% and 82% of proteins in Cp category of low and medium GC organisms, respectively). All the variables passed the Kolmogorov-Smirnov normality test. Q-Q plots of a normality test for disorder contents of proteins in Cp category of high and low GC organisms are represented on Figure 9. Similar plots may be produced for other categories of proteins and organisms.

In bacteria, high GC organisms are up to 100% more disordered than others; medium GC organisms are the least disordered.

These findings may be explained by a tendency of high GC value genomes toward increased propensity of Gly, Ala, Arg and Pro amino acids, which are also overexpressed in DPs [41,42].

Other characteristics (see the web site, link *L10*, for complete data) show the following relations to disorder:

#### genome size

While in archaea there is no difference between different genome sizes, in bacteria there is somewhat more disorder in high genome size organisms than in low size, by all the functional groups; results are in agreement with Foerstner K. U. et al. [41] i.e., with a tendency of large genomes to be GC rich (and consequently have a higher disorder) and small genomes to be GC poor.

#### oxygen requirement

Both archaea and bacteria have maximum disorder in aerobic organisms; results are in agreement with a conclusion of Naya H. et al. [43] that aerobiosis is strongly linked to a significant increment in GC% (and consequently have a higher disorder).

#### habitat

**Figure 10:**
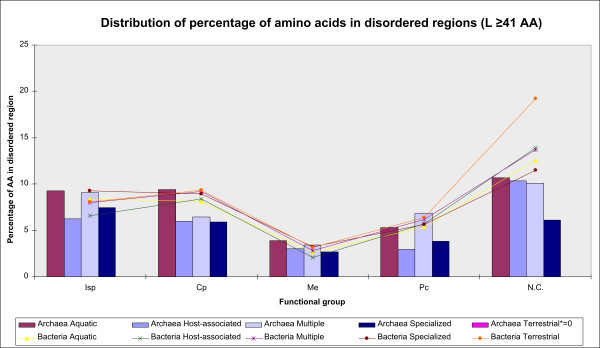
**Distribution of the percentage of amino acids in disordered regions by habitat modalities and COG functional groups**. Disordered regions of L ≥41 AA are considered.

In archaea, maximum disorder is found in aquatic organisms, by all the functional groups (Figure 10);

In bacteria, disorder is uniformly spread over habitat modalities, somewhat less in the Me functional group of host-associated organisms;

These results are in agreement with Mann S. & Chen Y-PP. [46] that free living organisms with larger genomes show a tendency toward higher GC content, as a result of a more complex and varied environments (and consequently have a higher disorder).

#### temperature range

In archaea, disorder level is higher in mesophilic organisms up to 200% as compared to other temperature ranges.

In bacteria, maximum disorder is in thermophilic organisms (except for Cp group of mesophilic organisms); hyperthermophilic organisms have less disorder than others.

As explained in Joshua L. C. [47] there is a tendency of organisms growing on higher temperature toward more stable protein folds (and less disorder content).

As far as it concerns phyla, in Euryarchaeota (Archaea) disorder is up to 3 times higher than in Crenarchaeota. In bacteria, the highest disorder is in high GC Bacteroidetes/Chlorobi organisms, by all the categories (as well as for regions of L ≥1, 11 AA), and in Bacteroidetes/Chlorobi specialized, aerobic organisms (habitat, oxygen); the least disorder is in the Me category of anaerobic Gammaproteobacteria. Bacteroidetes/Chlorobi high genome size organisms have lower level of disorder (below 50%) than low size genomes, in all the categories. As far as it concerns temperature, mesophilic Planctomycetes, and both mesophilic and thermophilic Deinococcus-Thermus and Actinobacteria have the highest disorder content.

### Cross correlation of ecological characteristics

Cross-correlations of all the pairs of characteristics with respect to disordered regions of L ≥1, 11, 41 AA, are presented on the web site, link *L11*. Table 6 represents cross correlation of *habitat *and *GC content *with respect to percentage of amino acids in disordered regions of L ≥41 AA, in different COG groups.

**Table 6:** Habitat and GC content with respect to disorder content.

				Functional group				
Superkingdom	Habitat	GC content	Type	Isp	Cp	Me	Pc	**N.C**.
Archaea	Aquatic	Low	disord	6,62	6,49	2,04	2,45	5,95
			order	58,38	64,00	69,7	69,30	62,37
		
		Medium	disord	6,17	5,30	1,82	3,59	6,25
			order	60,79	66,52	72,25	67,97	62,02
		
		High	disord	19,64	18,96	10,33	13,10	22,96
			order	36,88	43,07	52,41	50,50	39,54
	
	Host-associated	Low	disord	6,23	5,97	3,01	2,99	10,33
			order	58,03	64,74	65,43	67,49	63,06
	
	Multiple	Medium	disord	9,10	6,47	3,40	6,88	10,09
			order	56,44	59,63	66,73	59,45	51,53
	
	Specialized	Low	disord	4,01	1,23	0,80	1,69	4,52
			order	62,60	73,68	75,00	73,46	70,34
		
		Medium	disord	5,15	3,75	1,30	2,14	4,19
			order	60,24	69,05	72,27	70,48	68,23
		
		High	disord	14,61	12,93	7,59	8,79	11,49
			order	47,58	53,75	60,04	58,04	55,13

Bacteria	Aquatic	Low	disord	7,88	5,91	2,35	3,99	9,99
			order	55,10	61,05	67,17	65,13	52,94
		
		Medium	disord	8,03	8,03	2,45	5,19	11,66
			order	58,21	61,96	70,26	64,96	50,29
		
		High	disord	9,68	8,91	3,25	6,30	16,10
			order	56,24	61,49	69,10	63,55	49,63
	
	Host-associated	Low	disord	6,38	10,73	2,36	5,94	16,68
			order	60,97	59,47	69,75	64,57	49,09
		
		Medium	disord	5,72	6,85	1,44	4,89	10,39
			order	61,44	62,85	72,66	65,98	52,57
		
		High	disord	9,68	11,24	3,48	7,45	20,95
			order	57,97	59,96	70,02	63,99	46,55
	
	Multiple	Low	disord	6,17	7,63	2,17	5,78	10,35
			order	59,25	59,80	67,39	64,70	54,18
		
		Medium	disord	6,46	7,86	1,91	4,87	10,46
			order	60,14	61,65	71,09	65,52	52,75
		
		High	disord	10,43	11,21	4,06	7,94	17,98
			order	56,69	58,66	69,04	62,36	47,88
	
	Specialized	Low	disord	3,05	3,00	0,62	1,67	10,97
			order	73,33	77,47	80,73	78,30	82,12
		
		Medium	disord	6,93	6,89	1,91	4,36	8,36
			order	58,82	63,10	70,31	65,96	54,30
		
		High	disord	18,55	17,39	7,59	10,25	20,44
			order	45,73	50,16	61,44	56,42	44,65
	
	Terrestrial	Medium	disord	5,79	8,24	2,12	4,74	10,15
			order	58,47	59,00	68,56	64,75	52,53
		
		High	disord	10,23	10,34	4,29	8,36	27,54
			order	58,61	62,11	71,05	63,38	43,45

The percentage of amino acids in disordered regions of L ≥41 AA is represented for each pair of (habitat, GC content level), and each functional group of COGs (Cp, Isp, Me, Pc, N.C.), in archaea/bacteria.

The data in the table may be interpreted as follows:

In archaea, maximum disorder is found in the Isp group of aquatic, high GC organisms (19.64%), while the minimum is in the Me group of specialized, low GC organisms (0.80%). Percentage of disorder is always higher for high GC content organisms than for low and medium GC content organisms. Low and medium GC content organisms have similar disorder percentage for all the modalities of habitat. Independent-samples t-test suggests that (high GC, aquatic) and (high GC, specialized) organisms have significantly higher disorder contents than other combinations of (GC, habitat) (p < 0.01).

In bacteria, maximum disorder is found in the Isp group of specialized, high GC organisms (18.55%), while the minimum, the same as in archaea, in the Me group of specialized, low GC organisms (0.62%). Similar to archaea, high GC organisms have higher disorder content than low or medium GC organisms. Independent-samples t-test confirms significantly higher disorder contents of (high GC, specialized) organisms (p < 0.03).

Relations of other pairs of characteristics to disorder level are represented on the web site, link *L11*.

In general, it may be noticed that:

high GC organisms exhibit higher disorder than medium or low GC ones, as expected, since high GC increases frequency of Gly, Ala, Arg, Pro AA that are more represented in disordered regions of proteins (in agreement with [41,42];

COGs of proteins groups exhibit constant disorder ratio: Isp and Cp higher than Me, Isp higher than Cp in most cases (in agreement with [1,30].

The overall cross-correlation of pairs of characteristic with respect to disorder is represented in the Table 7.

**Table 7:** Cross-correlation of pairs of characteristics with respect to disorder.

	GC percent				Genome size			Oxygen requirement					Habitat						Temperature range			
									
	low	med	high		low	high		aero	facult	anaero	micro aero		aquat	host- assoc	mult	spec	terr		psych	meso	thermo	hyper thermo
**GC percent**																						
**low**					+	-		min	-	++	-		+	+	-	min	-		-	++	-	min
**medium**					min	++		+	+	++	-		+	-	+	+	-		-	++	+	+
**high**					max	-		+++	max	++	-		max	-	-	+++	-		-	max	-	++
																			

**Genome size**																						
**low**	+++	++	+++					max	-	min	-		max	min	-	+	-		-	max	min	+
**high**	-	++	max					-	-	+	-		++	-	+	-	-		-	++	-	-
																			

**Oxygen requirement**																						
**aerobic**	+	++	+++		++	max							max	-	-	+	-		-	+++	+	+
**facultative**	max	++	+++		+++	++							min	-	-	+++	-		-	max	+	min
**anaerobic**	+	min	++		++	min							+	+	+	++	-		-	++	++	++
**microaerophilic**	++	++	+++		++	++							-	-	-	-	-		-	-	-	-
																			

**Habitat**																						
**aquatic**	+	++	++		++	+++		+++	++	+	++								-	+++	-	+
**host-associated**	++	+	++		++	++		+++	++	min	++								-	++	-	min
**multiple**	++	++	+++		++	++		+++	++	++	+								-	++	-	-
**specialized**	min	++	max		++	min		max	+	+	-								-	max	+	+
**terrestrial**	-	++	+++		max	++		++	+	-	++								-	-	-	-
																			

**Temperature range**																						
**psychrophilic**	-	++	-		++	++		+	+	+	-		+	-	++	+	-					
**mesophilic**	++	++	max		++	max		++	++	+	+		++	++	+++	max	++					
**thermophilic**	-	++	+++		++	-		++	-	+	max		+	-	++	++	++					
**hyperthermophilic**	-	min	-		min	-		+	-	min	-		min	-	-	+	-					

Disorder level is measured by the percentage of amino acids in disordered regions of L≥41 AA in the proteomes of organisms with specific values (modalities) of pairs of characteristics. Maximum (max) and minimum (min) disorder content is identified for each pair of characteristics (e.g., GC content/genome size, GC content/oxygen requirement, etc.). Labels "+", "++", "+++" represent gradation in disorder content. Label "-" denotes an empty set of organisms. The North-East triangle corresponds to archaea, the South-West to bacteria.

### Association rules mined

**Figure 11:**
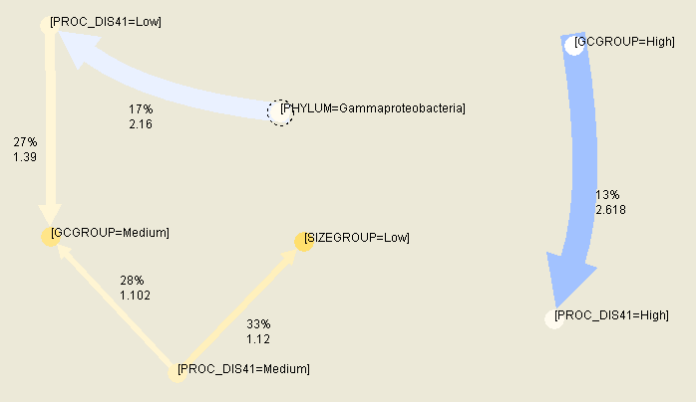
**The most reliable association rules for bacteria**. Parameter confidence is ≥66%, support ≥13%; PROC_DIS41 denotes percentage of amino acids in disordered regions of L ≥41 AA.

**Figure 12:**
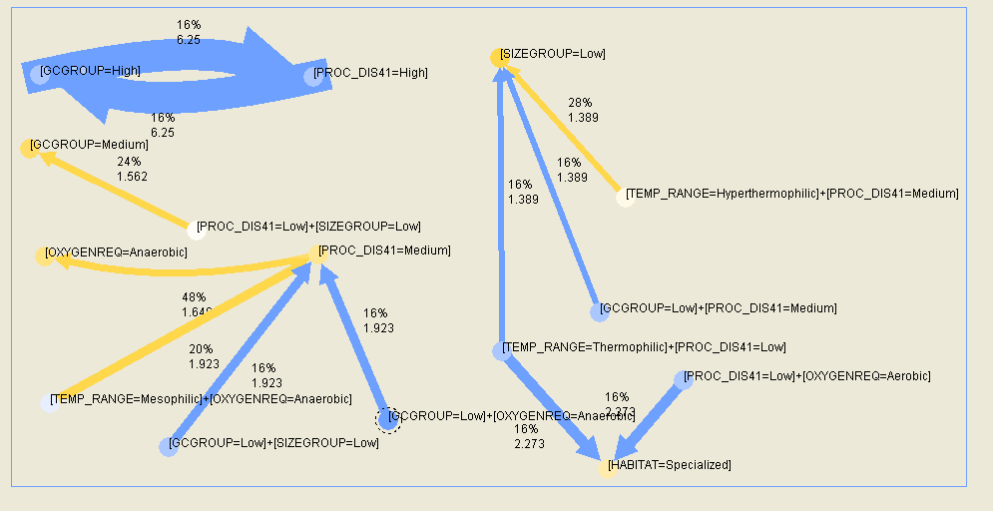
**The most reliable association rules for archaea**. Parameter confidence is ≥90%, support ≥16%; PROC_DIS41 denotes the percentage of amino acids in disordered regions of length ≥41 AA.

Results of association rule mining for bacteria and archaea are represented in Figure 11 and Figure 12. Each arrow is marked with support percentage and lift values. Thicker arrows correspond to higher lift, and color from blue to yellow corresponds to increasing support percentage.

Some of the most reliable rules for bacteria relate:

high GC content with high disorder (highest lift),

Gammaproteobacteria with low disorder (high lift)

medium disorder level with low genome size (high confidence, moderate support)

- both medium and low disorder level with medium GC content

Rules mined for archaea are in general more reliable than those for bacteria. Archaea are mostly anaerobic and this fact is reflected in the rules. For confidence higher than 90%, some of the most reliable rules relate:

medium disorder with anaerobic organisms (highest support),

high disorder with high GC content (highest lift)

Some less reliable rules mined relate low or medium disorder to low size genomes, low disorder thermophilic or aerobic archaea to specialized habitat, low GC low genome size or anaerobic organisms to medium disorder, low genome size low disorder to medium GC organisms.

## Conclusions

Exhaustive disorder proteins content analysis has been performed by functional classes of proteins, for a larger dataset of prokaryotic organisms than previously analyzed. Results obtained are well correlated to those previously published, with some extension in range of disorder level and clear distinction between functional classes of proteins. Wide correlation and association analysis between protein disorder and genomic and ecological characteristics has been performed for the first time. Obtained results give insights into multi-relationships among the main prokaryotic phenotypic characteristics and their protein disorder content. Such analyses provide useful hints for the better understanding of evolutionary processes and may be also useful for taxon determination.

The methods presented have their limitations. The main drawback of the approach is the fact that the disorder considered has been predicted and not experimentally established. Comparison and evaluation of different disorder predictors have shown that different predictors perform well for different types of disorder, and that new methods are necessary for recognizing different types of disorder, as well as regions undergoing disorder-to-order transition.

## Authors' contributions

GMP-L participated in the design and overall coordination of the study and drafted the methods and results parts of the manuscript. NSM recomputed all the parts and checked the results, produced final tables and figures, participated in manuscript finalizing, editing and formatting. JJK performed the computational analysis of disorder content, drafted tables and figures and participated in drawing figures. MVB studied literature, investigated biological impacts of the research, drafted the background part of the manuscript and participated in the design and overall coordination of the study. SNM designed and implemented the web site. ZO inspired the overall work and revised the final manuscript. All authors read and approved the final manuscript.

## Appendix

### Note added in proof

By the time our manuscript was reviewed (November 25th 2010), the NCBI prokaryotes database had been extended to 1363 sequenced organisms, about 63% COG annotated (as compared to the dataset we dealt with, as of November 20th 2009, containing 923 organisms of about 32% COG-annotated). We repeated some of the most important analyses on the new dataset: (1) the number of disordered regions per 100 AA by COGs and (2) average number of disordered regions per 100 AA by functional groups of COGs, for different region lengths. The results are generally stable, with differences below 10%, for disordered regions of unlimited length. The charts for these two measures have the same shapes as the corresponding ones for the original dataset (Figures 2, 8). We plan to repeat all the analyses performed and to extend the web site with the corresponding result data.

By the time we submitted the first version of our paper, a study has been published by Xue B. et al. (**Archaic chaos: intrinsically disordered proteins in Archaea**, *BMC Systems Biology*, 2010, **4(Suppl 1):**S1), that basically complements the methods applied and results presented in this paper.
